# Impact of dizziness on everyday life in older primary care patients: a cross-sectional study

**DOI:** 10.1186/1477-7525-9-44

**Published:** 2011-06-16

**Authors:** Jacquelien Dros, Otto R Maarsingh, Leo Beem, Henriëtte E van der Horst, Gerben ter Riet, François G Schellevis, Henk CPM van Weert

**Affiliations:** 1 Department of Family Medicine, Academic Medical Center, University of Amsterdam, Amsterdam, The Netherlands; 2 Department of Family Medicine and EMGO Institute for Health and Care Research, VU University Medical Center, Amsterdam, The Netherlands; 3 NIVEL, the Netherlands Institute for Health Services Research, Utrecht, The Netherlands

## Abstract

**Background:**

Dizziness is a common and often disabling symptom, but diagnosis often remains unclear; especially in older persons where dizziness tends to be multicausal. Research on dizziness-related impairment might provide options for a functional oriented approach, with less focus on finding diagnoses. We therefore studied dizziness-related impairment in older primary care patients and aimed to identify indicators related to this impairment.

**Methods:**

In a cross-sectional study we included 417 consecutive patients of 65 years and older presenting with dizziness to 45 general practitioners in the Netherlands from July 2006 to January 2008. We performed tests, including patient history, and physical and additional examination, previously selected by an international expert panel and based on an earlier systematic review. Our primary outcome was impact of dizziness on everyday life measured with the Dutch validated version of the Dizziness Handicap Inventory (DHI). After a bootstrap procedure (1500x) we investigated predictability of DHI-scores with stepwise backward multiple linear and logistic regressions.

**Results:**

DHI-scores varied from 0 to 88 (maximum score: 100) and 60% of patients experienced moderate or severe impact on everyday life due to dizziness. Indicators for dizziness-related impairment were: onset of dizziness 6 months ago or more (OR 2.8, 95% CI 1.7-4.7), frequency of dizziness at least daily (OR 3.3, 95% CI 2.0-5.4), duration of dizziness episode one minute or less (OR 2.4, 95% CI 1.5-3.9), presence of anxiety and/or depressive disorder (OR 4.4, 95% CI 2.2-8.8), use of sedative drugs (OR 2.3, 95% CI 1.3-3.8) , and impaired functional mobility (OR 2.6, 95% CI 1.7-4.2). For this model with only 6 indicators the AUC was .80 (95% CI .76-.84).

**Conclusions:**

Dizziness-related impairment in older primary care patients is considerable (60%). With six simple indicators it is possible to identify which patients suffer the most from their dizziness without exactly knowing the cause(s) of their dizziness. Influencing these indicators, if possible, may lead to functional improvement and this might be effective in patients with moderate or severe impact of dizziness on their daily lives.

## Background

Dizziness is one of the geriatric giants. Thirty percent of people over 65 years of age experience dizziness in some form [1-4], and this number increases to 50% in the very old (85+) [2]. Annual consultation rates for dizziness in primary care increase from 8% in patients over 65 years of age to 18% for the oldest elderly [5,6]. Besides, two-third of older dizzy patients experience persistent or recurrent dizziness for at least six months [3,7,8].

For physicians, older dizzy patients may be a challenge because of the wide range of underlying conditions. As dizziness in the elderly tends to be multicausal, it is often not possible to identify a specific etiological condition. Patients without a diagnosis make up 20-40% of all patients presenting with dizziness in general practice [9-11], and even if specific diseases are revealed, these cannot always be treated effectively. Nevertheless, dizziness can be extremely troublesome for older patients. It can lead to considerable impairment in daily functioning, and it is associated with social isolation, functional disability, falls, and with nursing home placement [4,8]. Accordingly, to adequately manage these patients, it is important to assess the impact of dizziness on everyday life experienced by older patients, and to identify factors modifying this impact on daily functioning. After ruling out serious conditions a functional oriented approach, with less focus on finding diagnoses, may be most beneficial to, especially older, patients. Such a functional approach has previously been suggested [4,6,12]. Influencing factors contributing to the impact of dizziness could lead to functional improvement and this might be most effective in patients with the highest impact of dizziness on their daily lives.

We therefore studied dizziness-related impairment in older primary care patients and identified factors related to this impairment.

## Methods

### Study design and participants

Every Dutch inhabitant is listed with a general practitioner (GP), and patients only consult a medical specialist after referral by their GP. In a cross-sectional study, between July 2006 and January 2008, 45 general practitioners (GPs) in 24 Dutch practices recruited consecutive patients aged at least 65 years who consulted for dizziness. We ensured consecutiveness by checking GPs' electronic medical records for missed inclusions each month.

Our definition of dizziness included patients describing a giddy or rotational sensation, a feeling of imbalance, light-headedness, and/or a sensation of impending faint. Criteria for exclusion were inability to speak Dutch or English, severe cognitive impairment, a corrected visual acuity of less than 3/60 for the best eye, impossibility of verbal communication, or wheelchair dependency. The study was approved by the medical ethics committees of both involved academic medical centers. All patients gave written informed consent.

### Definition of outcome

Our primary outcome was the impact of dizziness on everyday life, measured with the Dutch validated version of the Dizziness Handicap Inventory (DHI) [13,14] (additional file 1). The DHI is a self-report questionnaire used to assess the degree of disability associated with dizziness regardless of its underlying cause(s). The questionnaire contains 25 items covering three subscales with functional, emotional and physical aspects. "Yes" scores 4 points, "sometimes" 2 points and "no" 0 points. DHI-scores range from 0 to 100, higher scores indicating greater perceived disability. DHI-scores can be classified into mild (0-30 points), moderate (31-60 points), and severe (61-100 points) [15,16]. We included a 0/1 dichotomized DHI-score, with 1 representing scores greater than 30 (moderate or severe impact of dizziness).

The DHI is the mostly used questionnaire to quantify the impact of dizziness and has been translated to Swedish [17], Chinese [18], French [19], Dutch [14], Portuguese (Brazil) [20], German [21] and Norwegian [15]. High internal consistency and satisfactory test-retest reliability has been demonstrated for the total scale as well as in some studies for the subscales [13,16]. Other studies found similar results for the total scale, but questioned internal consistency of the subscales [15,22,23]. In summary, validity has been ascertained in secondary and tertiary care settings with mostly vertiginous patients in several studies [13-16,22-27].

### Indicators of impact of dizziness

In a 3-round Delphi procedure, 16 international experts, representing dizziness-relevant medical specialties, selected 21 tests feasible in primary care, and potentially contributing to the diagnostic process in older patients presenting with dizziness to a GP. The tests included four elements of patient history, eleven on physical examination, and six additional diagnostic tests [28,29]. In addition, we gathered information on demographic variables, and used the validated timed up-and-go test to measure functional mobility [30]. See for assessments of tests and measurements additional file 2.

From these tests and measurements resulted a total of 86 variables of which we selected 32 candidate indicators concerning demographic and lifestyle factors, characteristics of dizziness, data on relevant diagnoses and drugs, and information about relevant conditions or tests (e.g. orthostatic hypotension, functional mobility, Dix-Hallpike test). Inclusion criteria for this selection process were: (1) plausible relation with impact of dizziness, (2) for a GP easily to obtain information, (3) prevalence in the study population between 10% to 90%, and (4) Spearman correlation coefficient between -.50 and .50.

In the original dataset we imputed missing data using the iterative chained equations method (ICE) in STATA/SE 10.0 (StataCorp, College Station, TX, USA). Briefly, for each variable in turn missing values are filled in with random predicted values based on observed values. Then, filled-in values in the first variable are removed, leaving the original missing values for this variable. These missing values are then imputed using regression imputation on all other variables (inclusive their "filled-in" values). This process is repeated for each variable with missing values until one 'cycle' is completed. We continued this process for 5 cycles [31,32], and in this way 0.2% of all values in the original dataset were imputed [33].

### Statistical analyses

First, bivariate Pearson correlations of candidate indicators and DHI-scores were calculated to assess predictive performance of each indicator separately. Then predictability of continuous and dichotomous DHI-scores was investigated with multiple linear and logistic regressions. In each of 1500 bootstrap samples we used backward stepwise regression, starting with all variables in the model, which selected indicators for a more parsimonious model with good predictive performance. The selection criterion ("p-remove") was set at 0.05 and from the models selected in each bootstrap sample, variables were retained for a final model if they were selected in at least 67% of the 1500 samples (i.e. more than 1000). Next, the proportion of variance accounted for (R^2^) and Nagelkerke R^2 ^[34] were estimated for this final model. For comparison, we also calculated an average regression weight (B_m_) for each variable over all bootstrap samples, irrespective of the other variables selected in that particular sample. To obtain a weighted instead of a simple average, the regression weight in a sample was set to zero when a variable was not selected [35]. Odds ratios were calculated for the final logistic model with dichotomous DHI-scores. We calculated simple sum scores (presence indicator = 1, absence = 0) and weighted sum scores with the average regression weights for both final linear and logistic models.

The calibration of the logistic model was evaluated by comparing the observed and predicted outcome probabilities for all values of the simple sum score (0-6), and the fit was evaluated using the Hosmer-Lemeshow Goodness-of-Fit test. The ability of the logistic simple sum score model to discriminate between patients with high versus low impact of dizziness was estimated using the area under the Receiver Operating Characteristic (ROC) curve (AUC).

## Results

### Patient characteristics

Data were available from 417 older patients with dizziness (table 1) [29]. Their age ranged from 65 to 95 years with a mean age of 78.5 (SD = 7.1), 74% were female, and 69% experienced dizziness for at least six months.

**Table 1:** Patient characteristics of 417 dizzy older patients in primary care

	No. (%) of patients
Sex, female	307 (74)
Age in years, mean (range)	78.5 (65-95)
Living situation	
Alone	254 (61)
In residential home	66 (16)
Ethnic background	
Dutch native	342 (82)
Western immigrant	31 (7)
Non-western immigrant	44 (11)
Level of education	
Elementary school	119 (29)
High school	247 (59)
College/university	51 (12)
Medical history	
Cardiovascular disease	205 (49)
Hypertension	239 (57)
Diabetes	78 (19)
Neurologic disease	145 (35)
Psychiatric disease	142 (34)
Onset of dizziness	
<6 months	128 (31)
≥6 months	289 (69)
Category of dizziness*	
Disequilibrium	360 (86)
Presyncope	302 (72)
Vertigo	259 (62)
Atypical	146 (42)

*Adds up to more than 100%, because most patients described more than one subtype.

### Dizziness Handicap Inventory scores

The DHI-score varied from 0 to 88, with a median score of 34 and an interquartile range from 22 to 50 (additional file 3). A total of 182 patients (44%) were mildly disabled by their dizziness (score 0-30), 179 patients (43%) moderately (score 31-60), and 56 patients (13%) severely (score 61-100).

### Indicators of impact of dizziness

In univariate regression analysis the correlations between the impact of dizziness and candidate indicators were <0.3 for most factors. Only frequency of dizziness, functional mobility, and having an anxiety and/or depressive disorder had moderate correlations of 0.3 to 0.5.

#### Models with continuous and dichotomous DHI-scores (table 2 and table 3)

**Table 2:** Association of all candidate indicators with the impact of dizziness on everyday life in older primary care patients

	Prev, %	Linear Model (continuous DHI-scores)			Logistic Model (dichotomous DHI-scores)*			
		**P**_**1500**_	**B**_**m**_	**B**_**s**_	**P**_**1500**_	**B**_**m**_	OR (95%CI)	**B**_**s**_
**Demographic**								
Age		.09	.0.0		.11	0.0	1.0 (1.0-1.1)^§^	
Sex, female	74	.52	2.7		.35	0.2	1.8 (1.2-2.8)	
Ethnicity, non-western	7	.08	0.4		.09	0.1	1.0 (0.5-2.2)	
Living in residential home	16	.23	1.2		.09	0.2	2.1 (1.2-3.7)	
**Lifestyle factors**								
Smoking	15	.06	0.2		.46	0.5	1.3 (0.7-2.2)	
Excessive alcohol intake	7	.06	0.4		.07	0.0	0.6 (0.3-1.3)	
**Dizziness characteristics**								
Onset, 6 months ago or more	**69**	**.94**	**5.9**	**7.3**	**.92**	**1.0**	**2.6 (1.7-4.1)**	**1.04**
Frequency, at least daily	**57**	**1.00**	**9.3**	**10.5**	**.97**	**1.1**	**2.9 (1.9-4.3)**	**1.20**
Duration, one minute or less	**45**	**.96**	**6.2**	**7.7**	**.89**	**1.0**	**0.4 (0.3-0.6)**	**.89**
*Subtype description of dizziness*								
Light-headedness/presyncope	72	.08	-0.2		.07	0.0	1.2 (0.8-1.9)	
Spinning sensation/vertigo	62	.06	0.1		.07	0.1	1.1 (0.8-1.7)	
Unsteadiness/disequilibrium	86	.30	1.9		.30	0.1	3.0 (1.7-5.4)	
Not classifiable dizziness	42	.06	-0.1		.18	0.2	1.5 (1.0-2.3)	
*Provoking circumstances*								
Standing still	24	.62	3.4		.36	0.4	3.1 (1.9-5.1)	
Exercise	31	.21	0.8		.25	0.2	1.5 (1.0-2.2)	
Changes in head position	79	.31	1.7		.38	0.5	2.5 (1.5-4.0)	
Getting up from lying or sitting	70	.11	0.4		.06	0.0	1.6 (1.1-2.5)	
*Associated symptoms*								
Presyncopal symptoms (without panic disorder)	41	.44	2.1		.10	0.0	1.3 (0.9-1.9)	
Trouble with walking and/or (almost) falling	57	.46	2.3		.47	0.4	3.0 (2.0-4.5)	

**Table 3:** Association of all candidate indicators with the impact of dizziness on everyday life in older primary care patients

	Prev, %	Linear Model (continuous DHI-scores)			Logistic Model (dichotomous DHI-scores)*			
		**P**_**1500**_	**B**_**m**_	**B**_**s**_	**P**_**1500**_	**B**_**m**_	OR (95%CI)	**B**_**s**_
**Relevant diseases and drugs**								
Cardiovascular disease	85	.05	0.0		.14	-0.2	1.6 (0.9-2.7)	
Diabetes	19	.07	0.2		.15	0.0	1.4 (0.8-2.3)	
Hearing problems	73	.20	0.9		.50	0.5	2.2 (1.4-3.4)	
Anxiety and/or depressive disorder	**22**	**1.00**	**11.0**	**12.6**	**.95**	**1.2**	**7.2 (3.8-13.7)**	**1.48**
Poly-pharmacy	42	.41	1.9		.55	0.6	2.3 (1.6-3.5)	
Use of sedative drugs	**31**	**.95**	**6.3**	**7.0**	**.71**	**0.7**	**2.9 (1.8-4.6)**	**.82**
**Information relevant conditions or tests**								
Often unexplained complaints	15	.41	2.5		.08	0.1	2.0 (1.1-3.7)	
Orthostatic hypotension	24	.26	-1.2		.11	0.0	1.3 (0.8-2.1)	
Functional mobility	**60**	**.97**	**7.2**	**8.2**	**.91**	**1.2**	**4.0 (2.6-6.0)**	**.97**
Impairment of hip/knee/ankle joints	51	.21	-0.9		.08	0.0	1.8 (1.2-2.6)	
Neurological impairment feet	65	.19	-0.8		.15	-0.2	1.2 (0.8-2.8)	
Dix-Hallpike test	12	.50	3.6		.26	0.4	1.5 (0.8-2.8)	
Visual acuity	29	.29	1.3		.17	0.2	1.7 (1.1-2.7)	

Stepwise backward linear and logistic regression analysis, bootstrap 1500x, α = .05. Variables selected in ≥1000 of the 1500 bootstrap samples were retained for the final models and highlighted in bold (indicators).

Prev: prevalence in the research population; B_m_: average regression weight over all bootstrap samples; B_s_: regression weight in selected model; OR: Odds Ratio; CI: Confidence Interval. ^§^OR is estimated per year increase or decrease.*Dichotomous DHI-scores: scores 0-30 (mild impact of dizziness) = 0, scores 31-100 (moderate or severe impact of dizziness) = 1.

Indicators retained in the model after our selection were similar for continuous and dichotomous DHI-scores: (1) onset of dizziness (6 months ago or more), (2) frequency of dizziness (at least daily), (3) duration of dizziness episode (one minute or less), (4) anxiety and/or depressive disorder, (5) use of sedative drugs, and (6) (impaired) functional mobility measured with the timed up-and-go test.

All correlations between the variables were weak (correlation coefficients <0.3), confirming that these factors represented different independent relations to the DHI. For the continuous DHI, the R^2 ^was .40 in the model with 6 indicators, compared to .46 for the model with all variables. This means that, concerning the impact of dizziness, only little information was lost using six indicators versus all variables. Where the R^2 ^of the weighted sum score for the 6 indicators was .40, the R^2 ^of the simple sum score was .39, indicating that little information was lost in using the simple sum score. For the dichotomous DHI, the Nagelkerke R^2 ^with 6 indicators was .37, compared to .45 for the model with all variables. The R^2 ^of the simple sum score was as good as the R^2 ^of the weighted sum score, both .37.

**Figure 1:**
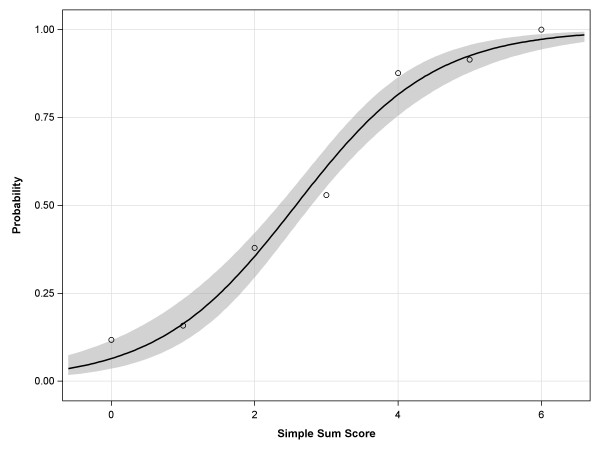
**Observed and predicted probabilities of experiencing moderate or severe impact of dizziness (DHI > 30) for all values of the simple sum score (0-6) of indicators**. o: proportion of observed dizziness impact (DHI > 30) corresponding with that particular sum score; **—** : proportion of predicted dizziness impact (DHI > 30); the grey band represents the 95% confidence interval. A simple sum score of ≥4 means a probability of ≥.80 that an older patient experiences moderate or severe impact of dizziness on everyday life.

**Figure 2:**
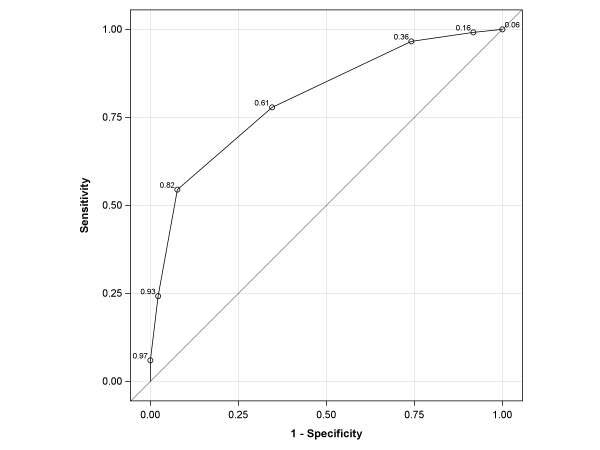
**ROC curve of the final logistic model with six indicators related to the impact of dizziness on everyday life of older primary care patients**. Area Under the Curve (AUC) is .80 (CI .76-.84). In the ROC curve the predicted probabilities for all values of the sum score and their corresponding sensitivity and (1-)specificity. The predicted probability of 0.82 corresponds with a simple sum score of 4.

Figure 1 shows the proportions of observed and expected impact of dizziness (DHI > 30) for all values of the simple sum score. The Hosmer-Lemeshow test (p = .16) indicated that the observed impact of dizziness (DHI > 30) matched the expected impact of dizziness for the simple sum scores, confirming the reliability and the goodness-of-fit of the predictability of the logistic model. Figure 2 shows the ROC-curve of the final logistic model with an AUC of .80 (95% CI = .76 to .84).

## Discussion

This is one of the few published studies that address the impact of dizziness on everyday life in older primary care patients. Several studies reported on the impact of dizziness, of which some in older patients, but mostly in secondary and/or tertiary care settings with highly selected patients [23,36-38]. Other studies focussed on the impact of dizziness in home-dwelling ambulant, older, persons not presenting with, but asked for complaints of dizziness [8,39-41].

Frequency of attacks and psychological distress by anxiety and/or depression were also found to be major determinants of perceived impairment in older Chinese patients with chronic dizziness [38]. In a Swedish study in a 76-year-old home-dwelling ambulant population impairment increased with the number of attacks, but duration of dizziness showed no clear trend [39]. In a general practice community sample of working age people anxiety was associated with higher levels of dizziness-related impairment [8]. Other studies found significantly more dizziness-related impairment in participants with than without vestibular symptoms [37,41]. This factor was not found to be related in our study, but differences in the study populations could be due to this: in Gopinath's study 'older' was defined as aged ≥49 years (in our study ≥65), and in Neuhauser's study participants were aged 18 years or older. The prevalence of specific symptoms according the categories presyncope, vertigo, disequilibrium and atypical dizziness differs according to age: in younger patients (<40) atypical dizziness and presyncope prevail, in the middle aged (40-65) vertigo is the most prominent, and in the elderly (>65) presyncope and disequilibrium are more prevalent. In our study we could not ascertain associations with any dizziness category and this reflects the fact that, in particular elderly patients' dizziness cannot always be placed in one category. Besides, in both above mentioned studies participants were not presenting with, but were asked for complaints of dizziness. This selection method may give an overrepresentation of vertigo, knowing that vertiginous dizziness is more easily recognized as dizziness by participants.

### Strengths and limitations of the study

An important strength of our study is that we were quite complete in assessing the contribution of potentially relevant indicators by choosing variables from a great spectrum of the diagnostic process, including demographic data, history, physical examination and diagnostic tests. In spite of this, some potential indicators may have been missed. For example, we did not ask about recent falls. Another strength of this study is our sampling procedure with which we ensured the inclusion of consecutive patients to rule out selection bias.

This study has several limitations. First, the generalizability of our findings might be limited to older primary care patients. A second limitation is the observational cross-sectional design itself. Although we identified clinical plausible indicators, of which some have been associated with dizziness-related impairment in previous studies as discussed above, these show associations and not causality. However, although cause-effect relationships cannot be determined from this cross-sectional study, our findings, like stated by others [4,6,12], suggest that it would be interesting to try to reduce dizziness-related impairment by influencing treatable associated factors.

Another limitation concerns the DHI which has also been criticised [24]. Criticism on the various validation studies of the DHI is about the overrepresentation of chronic dizzy patients, with symptoms lasting ≥6 months. In our study two-third of the patients experienced dizziness for at least six months, which might suggest that the overrepresentation of chronic dizziness in an older population is apparently representative.

## Conclusions

Almost 60% of dizzy older primary care patients experience moderate or severe impact on everyday life due to dizziness. We identified six factors indicating which patients suffer the most from their dizziness without exactly knowing the cause(s) of their dizziness. These all include easily to obtain information, with certain features of dizziness (chronic dizziness (≥6 months), frequency at least daily, and duration of dizziness (≤1 minute)), having an anxiety and/or depressive disorder, the use of sedative drugs (mainly benzodiazepines), and poor functional mobility. A GP can identify these indicators within a few minutes and could taper treatment according the presence of these indicators, thereby focusing on interventions that might reduce the impact of dizziness on functional disability.

Future research is needed to analyse the predictive value of these and other indicators, which may provide a framework for effective dizziness management and give direction to diagnoses of dizziness and treatment options.

## List of abbreviations

AUC: area under the ROC curve; B_m_: average regression weight; B_s_: regression weight in the selected model; CI: confidence interval; DHI: Dizziness Handicap Inventory; GP: general practitioner; ICE: iterative chained equations method; OR: odds ratio; R^2^: proportion of variance; ROC: receiver operating characteristic.

## Competing interests

The authors declare that they have no competing interests.

## Authors' contributions

JD designed and carried out the study, performed the statistical analyses, drafted and wrote the manuscript. OM contributed substantially to the acquisition of data, helped to interpret results, and to draft and write the manuscript. LB performed the statistical analyses, helped to interpret results, and to draft the manuscript. HvdH helped to interpret results, and to draft the manuscript. GtR participated in the design of the study, performed part of the statistical analyses (imputation), helped to interpret results, and to draft the manuscript. FS helped to interpret results, and to draft the manuscript. HvW conceived the study, participated in its design and coordination, helped to interpret results, and draft the manuscript. All authors read and approved the final version.

## Supplementary Material

Additional file 1 Dizziness Handicap Inventory, the original version by Jacobson and Newman [13]. Click here for file

Additional file 2 Assessments of tests and measurements. Click here for file

Additional file 3 DHI-scores and estimated kernel density curve. Click here for file

## References

[B1] Colledge N Wilson JA Macintyre CCA MacLennan WJ The prevalence and characteristics of dizziness in an elderly community Age & Ageing 1994 23 117 120 10.1093/ageing/23.2.117 8023718

[B2] Jonsson R Sixt E Landahl S Rosenhall U Prevalence of dizziness and vertigo in an urban elderly population J Vestib Res 2004 14 47 52 15156096

[B3] Sloane P Blazer D George LK Dizziness in a community elderly population J Am Geriatr Soc 1989 37 101 108 2783432 10.1111/j.1532-5415.1989.tb05867.x

[B4] Tinetti ME Williams CS Gill TM Dizziness among older adults: A possible geriatric syndrome Annals of Internal Medicine 2000 132 337 344 10691583 10.7326/0003-4819-132-5-200003070-00002

[B5] Maarsingh OR Dros J Schellevis FG van Weert HC Bindels PJ van der Horst HE Dizziness reported by older patients in family practice: prevalence, incidence, and clinical characteristics BMC Fam Pract 2010 11 2 10.1186/1471-2296-11-2 20064231 PMC2817676

[B6] Sloane PD Coeytaux RR Beck RS Dallara J Dizziness: State of the science Annals of Internal Medicine 2001 134 823 832 11346317 10.7326/0003-4819-134-9_part_2-200105011-00005

[B7] Kruschinski C Klaassen A Breull A Broll A Hummers-Pradier E Priorities of elderly dizzy patients in general practice. Findings and psychometric properties of the "Dizziness Needs Assessment" (DiNA) Z Gerontol Geriatr 2010 43 317 323 10.1007/s00391-010-0098-5 20198376

[B8] Yardley L Owen N Nazareth I Luxon L Prevalence and presentation of dizziness in a general practice community sample of working age people Br J Gen Pract 1998 48 1131 1135 9667086 PMC1410052

[B9] Hanley K O'Dowd T Symptoms of vertigo in general practice: a prospective study of diagnosis Br J Gen Pract 2002 52 809 812 12392120 PMC1316083

[B10] Kroenke K Lucas CA Rosenberg ML Scherokman B Herbers JE Jr Wehrle PA Causes of persistent dizziness. A prospective study of 100 patients in ambulatory care Annals of Internal Medicine 1992 117 898 904 1443950 10.7326/0003-4819-117-11-898

[B11] Lawson J Fitzgerald J Birchall J Aldren CP Kenny RA Diagnosis of geriatric patients with severe dizziness Journal of the American Geriatrics Society 1999 47 12 17 9920224 10.1111/j.1532-5415.1999.tb01895.x

[B12] Kao AC Nanda A Williams CS Tinetti ME Validation of dizziness as a possible geriatric syndrome Journal of the American Geriatrics Society 2001 49 72 75 10.1046/j.1532-5415.2001.49012.x 11207845

[B13] Jacobson GP Newman CW The development of the Dizziness Handicap Inventory Arch Otolaryngol Head Neck Surg 1990 116 424 427 2317323 10.1001/archotol.1990.01870040046011

[B14] Vereeck L Truijen S Wuyts F Van de Heyning PH Test-retest reliability of the Dutch version of the Dizziness Handicap Inventory B-ENT 2006 2 75 80 16910291

[B15] Tamber AL Wilhelmsen KT Strand LI Measurement properties of the Dizziness Handicap Inventory by cross-sectional and longitudinal designs Health Qual Life Outcomes 2009 7 101 10.1186/1477-7525-7-101 20025754 PMC2804706

[B16] Whitney SL Wrisley DM Brown KE Furman JM Is perception of handicap related to functional performance in persons with vestibular dysfunction? Otology & Neurotology 2004 25 139 143 10.1097/00129492-200403000-00010 15021773

[B17] Jarlsäter S Mattsson E Test of reliability of the Dizziness Handicap Inventory and the Activities-specific Balance Confidence Scale for use in Sweden Adv Physiother 2003 5 137 144 10.1080/14038190310004385

[B18] Poon DM Chow LC Au DK Hui Y Leung MC Translation of the dizziness handicap inventory into Chinese, validation of it, and evaluation of the quality of life of patients with chronic dizziness Ann Otol Rhinol Laryngol 2004 113 1006 1011 15633905 10.1177/000348940411301212

[B19] Nyabenda A Briart C Deggouj N Gersdorff M Normative study and reliability of French version of the dizziness handicap inventory Ann Readapt Med Phys 2004 47 105 113 15059673 10.1016/j.annrmp.2003.11.002

[B20] Castro AS Gazzola JM Natour J Gananca FF Brazilian version of the dizziness handicap inventory Pro Fono 2007 19 97 104 17461352 10.1590/s0104-56872007000100011

[B21] Kurre A van Gool CJ Bastiaenen CH Gloor-Juzi T Straumann D de Bruin ED Translation, cross-cultural adaptation and reliability of the german version of the dizziness handicap inventory Otol Neurotol 2009 30 359 367 10.1097/MAO.0b013e3181977e09 19225437

[B22] Asmundson GJ Stein MB Ireland D A factor analytic study of the dizziness handicap inventory: does it assess phobic avoidance in vestibular referrals? J Vestib Res 1999 9 63 68 10334018

[B23] Perez N Garmendia I Garcia-Granero M Martin E Garcia-Tapia R Factor analysis and correlation between Dizziness Handicap Inventory and Dizziness Characteristics and Impact on Quality of Life scales Acta Otolaryngol Suppl 2001 545 145 154 11677730 10.1080/000164801750388333

[B24] Duracinsky M Mosnier I Bouccara D Sterkers O Chassany O Literature review of questionnaires assessing vertigo and dizziness, and their impact on patients' quality of life Value Health 2007 10 273 284 10.1111/j.1524-4733.2007.00182.x 17645682

[B25] Enloe LJ Shields RK Evaluation of health-related quality of life in individuals with vestibular disease using disease-specific and general outcome measures Phys Ther 1997 77 890 903 9291947 10.1093/ptj/77.9.890

[B26] Fielder H Denholm SW Lyons RA Fielder CP Measurement of health status in patients with vertigo Clin Otolaryngol Allied Sci 1996 21 124 126 10.1111/j.1365-2273.1996.tb01314.x 8735395

[B27] Jacobson GP Newman CW Hunter L Balzer GK Balance function test correlates of the Dizziness Handicap Inventory J Am Acad Audiol 1991 2 253 260 1837740

[B28] Maarsingh OR Dros J van Weert HC Schellevis FG Bindels PJ van der Horst HE Development of a diagnostic protocol for dizziness in elderly patients in general practice: a Delphi procedure BMC Fam Pract 2009 10 12 10.1186/1471-2296-10-12 19200395 PMC2660288

[B29] Maarsingh OR Dros J Schellevis FG van Weert HC van der Windt DA ter Riet G van der Horst HE Causes of persistent dizziness in elderly patients in primary care: a diagnostic study based on panel diagnosis Annals of Family Medicine 2010 8 196 205 10.1370/afm.1116 20458102 PMC2866716

[B30] Podsiadlo D Richardson S The timed 'Up and Go': A test of basic functional mobility for frail elderly persons Journal of the American Geriatrics Society 1991 39 142 148 1991946 10.1111/j.1532-5415.1991.tb01616.x

[B31] Buuren van S Boshuizen HC Knook DL Multiple imputation of missing blood pressure covariates in survival analysis Stat Med 1999 18 681 694 10.1002/(SICI)1097-0258(19990330)18:6<681::AID-SIM71>3.0.CO;2-R 10204197

[B32] Royston P Multiple imputation of missing values: Update of ice The Stata Journal 2005 5 527 536

[B33] Dros J Maarsingh OR van der Windt DA Oort FJ Ter Riet G de Rooij SE Schellevis F van der Horst HE van Weert HC Profiling dizziness in older primary care patients: an empirical study PLoS One 2011 6 e16481 10.1371/journal.pone.0016481 21304984 PMC3031582

[B34] Nagelkerke NJD A note on a general definition of the coefficient of determination Biometrika 1991 78 691 692 10.1093/biomet/78.3.691

[B35] Schomaker M Wan ATK Heumann C Frequentist Model Averaging with missing observations Computational Statistics & Data Analysis 2010 54 3336 3347 10.1016/j.csda.2009.07.02321696381

[B36] Bronstein AM Golding JF Gresty MA Mandala M Nuti D Shetye A Silove Y The social impact of dizziness in London and Siena J Neurol 2010 257 183 190 10.1007/s00415-009-5287-z 19701661

[B37] Gopinath B McMahon CM Rochtchina E Mitchell P Dizziness and vertigo in an older population: the Blue Mountains prospective cross-sectional study Clin Otolaryngol 2009 34 552 556 10.1111/j.1749-4486.2009.02025.x 20070765

[B38] Hsu LC Hu HH Wong WJ Wang SJ Luk YO Chern CM Quality of life in elderly patients with dizziness: analysis of the Short-Form Health Survey in 197 patients Acta Otolaryngol 2005 125 55 59 10.1080/00016480410017512 15799575

[B39] Grimby A Rosenhall U Health-related quality of life and dizziness in old age Gerontology 1995 41 286 298 10.1159/000213696 8537013

[B40] Lasisi AO Gureje O Disability and quality of life among community elderly with dizziness: report from the Ibadan Study of Ageing J Laryngol Otol 2010 1 6 10.1017/S0022215110000538 PMC4950960 20307358

[B41] Neuhauser HK Radtke A von BM Lezius F Feldmann M Lempert T Burden of dizziness and vertigo in the community Arch Intern Med 2008 168 2118 2124 10.1001/archinte.168.19.2118 18955641

