# Supplementary material for: Impact of dizziness on everyday life in older primary care patients: a cross-sectional study
Source: Health Qual Life Outcomes. 2011 Jun 16;9:44. doi: 10.1186/1477-7525-9-44 (PMC3142198; doi:10.1186/1477-7525-9-44)
Supplement: Additional file 1 — Dizziness Handicap Inventory, the original version by Jacobson and Newman [13]. [file 1477-7525-9-44-S1.DOC]

# Additional file 1 Dizziness Handicap Inventory, English version

**Date** (dd/mm/yyyyj) **__ __ / __ __ / 200__**

| **Sub-scale*** | **DIZZINESS HANDICAP INVENTORY: Put an ‘X’ for the most suitable answer. Try to fill in the form completely.** | **Yes** | **Some-times** | **No** |
| --- | --- | --- | --- | --- |
| P | 1. Does looking up increase your problem? |  |  |  |
| E | 2. Because of your problem, do you feel frustrated? |  |  |  |
| F | 3. Because of your problem, do you restrict your travel for business or recreation? |  |  |  |
| P | 4. Does walking down the aisle of a supermarket increase your problem? |  |  |  |
| F | 5. Because of your problem, do you have difficulty getting into or out of bed? |  |  |  |
| F | 6. Does your problem significantly restrict your participation in social activities such as going out to dinner, going to movies, dancing, or to parties? |  |  |  |
| F | 7. Because of your problem, do you have difficulty reading? |  |  |  |
| P | 8. Does performing more ambitious activities like sports, dancing, household chores such as sweeping or putting dishes away increase your problem? |  |  |  |
| E | 9. Because of your problem, are you afraid to leave your home without having someone accompany you? |  |  |  |
| E | 10. Because of your problem, have you been embarrassed in front of others? |  |  |  |
| P | 11. Do quick movements of your head increase your problem? |  |  |  |
| F | 12. Because of your problem, do you avoid heights? |  |  |  |
| P | 13. Does turning over in bed increase your problem? |  |  |  |
| F | 14. Because of your problem, is it difficult for you to do strenuous housework or yard work? |  |  |  |
| E | 15. Because of your problem, are you afraid people may think you are intoxicated? |  |  |  |
| F | 16. Because of your problem, is it difficult for you to walk by yourself? |  |  |  |
| P | 17. Does walking down a sidewalk increase your problem? |  |  |  |
| E | 18. Because of your problem, is it difficult for you to concentrate? |  |  |  |

*Subscales: F=functional, E=emotional, P=physical Jacobson and Newman, 1990

| **Sub-scale*** | **DIZZINESS HANDICAP INVENTORY: Put an ‘X’ for the most suitable answer. Try to fill in the form completely.** | **Yes** | **Some-times** | **No** |
| --- | --- | --- | --- | --- |
| F | 19. Because of your problem, is it difficult for you to walk around your house in the dark? |  |  |  |
| E | 20. Because of your problem, are you afraid to stay home alone? |  |  |  |
| E | 21. Because of your problem, do you feel handicapped? |  |  |  |
| E | 22. Has your problem placed stress on your relationships with members of your family or friends? |  |  |  |
| E | 23. Because of your problem, are you depressed? |  |  |  |
| F | 24. Does your problem interfere with your job or household responsibilities? |  |  |  |
| P | 25. Does bending over increase your problem? |  |  |  |
