# Supplementary material for: Impact of dizziness on everyday life in older primary care patients: a cross-sectional study
Source: Health Qual Life Outcomes. 2011 Jun 16;9:44. doi: 10.1186/1477-7525-9-44 (PMC3142198; doi:10.1186/1477-7525-9-44)
Supplement: Additional file 2 — Assessments of tests and measurements. [file 1477-7525-9-44-S2.DOC]

**Additional file 2**

**DIEP Evaluation**

All subjects underwent a standardized, comprehensive evaluation based on a set of diagnostic tests that were developed during an international Delphi procedure [28]. The patients were examined in a family practice setting by two study authors (JD, OM) and 3 well-trained physicians. Patients not able to visit the practice for medical reasons were examined at home.

*Medical History*

We recorded socio-demographic characteristics (including age, sex, ethnicity, education, and living situation), smoking habits, alcohol intake, current medication use (including the presence of polypharmacy, the use of fall-risk-inducing drugs (FRID), and the number of FRID per patient), medical history (including 26 yes/no questions about diseases frequently associated with dizziness), characteristics of dizziness (including description, onset, duration, frequency, provoking circumstances, and associated symptoms), and the use of a hearing, seeing, or walking aid. All subjects had to complete the Primary Care Evaluation

of Mental Disorders (PRIME-MD) Patient Health Questionnaire (PHQ), a self-administered instrument to assess psychiatric disorders, and the Dizziness Handicap Inventory (DHI), a questionnaire quantifying dizziness-related impact on everyday life[13,14].

*Physical examination*

Diagnostic criteria of measurements and tests are displayed in the table below.

Cardiovascular examination consisted of measurement of pulse and blood pressure, the orthostatic hypotension test, and auscultation of the heart. For measuring blood pressure we used a calibrated Omron M6 Blood Pressure Monitor. For the orthostatic hypotension test we measured blood pressure after 5 minutes in supine position, then measured it again in a standing position after 1, 2, 3, 4, and 5 minutes. The reproduction of symptoms was registered, but was not part of the definition. Additional cardiovascular assessment was performed on indication. Electrocardiography (ECG) was indicated if (1) a patient complained of presyncopal dizziness (faint feeling, lightheadedness, or feeling everything turning black), (2) dizziness was accompanied by palpitations, or (3) exercise provoked complaints of dizziness. For a digital 12-lead ECG we used a Welch Allyn Cardio PerfectTM Workstation. The ECGs were interpreted by two experienced cardiologists. Patient-activated continuous-loop event recording (CER) was indicated if an ECG did not indicate a clear explanation. For CER we used a Card Guard CG-6106 loop recorder. This two-lead ECG recorder was programmed to store 30 seconds before and 2 minutes after activation by the patient, with a maximum of four separate registrations. We sent the recordings to a specialized medical centre (Hartis B.V.), where they were interpreted by trained physicians. For financial reasons, we only performed CER for the first 60 consecutive patients.

Evaluation of the locomotor system consisted of orthopaedic screening of the lower limbs (stability in rest and during walking, range of movement of hips, knees, and ankles), tandem gait, and the timed up-and-go test (a test used to quantify functional mobility). Neurological evaluation consisted of examination of tendon reflexes, and the Semmes-Weinstein monofilament test (5.07/10g) to detect peripheral neuropathy. Evaluation of the vestibular system consisted of otoscopy, the Dix-Hallpike manoeuvre, and audiometry. For audiometry we used a Welch Allyn AM 232TM Manual Audiometer. Visual acuity was measured by means of a well-lit eye chart with Landolt rings. Laboratory evaluation included the measurement of hemoglobin level and non-fasting blood glucose. We used a Hemocue 201 analyzer for the measurement of haemoglobin levels and an Ascensia Breeze Glucose Meter for blood glucoses.

| **Diagnostic Criteria During the Evaluation of Dizzy Elderly Patients** | |
| --- | --- |
| **System** | **Diagnostic Criteria** |
| Cardiovascular system | |
| Bradycardia | <60 beats per minute, and <50 beats per minute when taking β-blockers |
| Tachycardia | >100 beats per minute |
| Elevated blood pressure | Systolic blood pressure 160 mm Hg after 5 min in supine position |
| Positive orthostatic hypotension test | A 20-mm Hg decrease in systolic blood pressure, or a systolic blood pressure below 90 mm Hg, measured at any of the given time periods (after 1, 2, 3, 4, or 5 minutes in standing position) |
| Explanatory abnormalities during ECG or CER | Bradycardia, tachycardia, atrial fibrillation/flutter, supraventricular tachycardia, ventricular tachycardia, ventricular fibrillation, torsades de pointes, brady-tachy syndrome, or a 3rd-degree atrioventricular block |
| Locomotor system | |
| Abnormal tandem gait | A tandem gait score of 40 seconds or more^a^ |
| Abnormal timed up-and-go test | A timed up-and-go score of 20 seconds or more |
| Neurological system | |
| Abnormal patellar tendon reflex | Clonus, or absent reflex |
| Abnormal Achilles tendon reflex | Clonus, or absent reflex |
| Abnormal plantar responses | Sign of Babinski |
| Peripheral neuropathy | The inability to perceive a monofilament (Semmes-Weinstein monofilament test) for at least 2 of 8 anatomical sites |
| Vestibular system | |
| Abnormal otoscopy | Otitis media, otitis externa, perforated tympanic membrane, or deformities tympanic membrane caused by trauma or surgery |
| Positive Dix-Hallpike maneuver | Vertigo associated with a mixed torsional and vertical nystagmus, a decline in the provoked vertigo and nystagmus within 30 seconds, and fatigability if the test was repeated |
| Abnormal audiometry | A Fletcher index of at least 35 dB for both ears^b^ |
| Laboratory tests | |
| Moderately severe anemia | Hemoglobinlevels between 5.0-6.0 mmol/L (8.1-9.7 g/dL) in women, or between 5.0-6.5 mmol/L (8.1-10.5 g/dL) in men |
| Severe anemia | Hemoglobin levels of <5.0 mmol/L (<8.1 g/dL) |
| Hyperglycemia | A nonfasting blood glucose of >11.0 mmol/L (>200 mg/dL) |
| Hypoglycemia | A nonfasting blood glucose of <3.0 mmol/L (<55 mg/dL) |
| Other | |
| Impaired vision | Corrected visual acuity of ODS ≤0.5 |
| CER = patient-activated continuous-loop event recording; ECG = electrocardiography; ODS = oculi dexter et sinister.  ^a^ Tandem gait score: the time in seconds added to the number of mistakes, multiplied by 3.  ^b^ Fletcher index: the average hearing loss in decibels for 1, 2, and 4 kHz. | |
