# Supplementary figures and images for: Impact of dizziness on everyday life in older primary care patients: a cross-sectional study
Source: Health Qual Life Outcomes. 2011 Jun 16;9:44. doi: 10.1186/1477-7525-9-44 (PMC3142198; doi:10.1186/1477-7525-9-44)

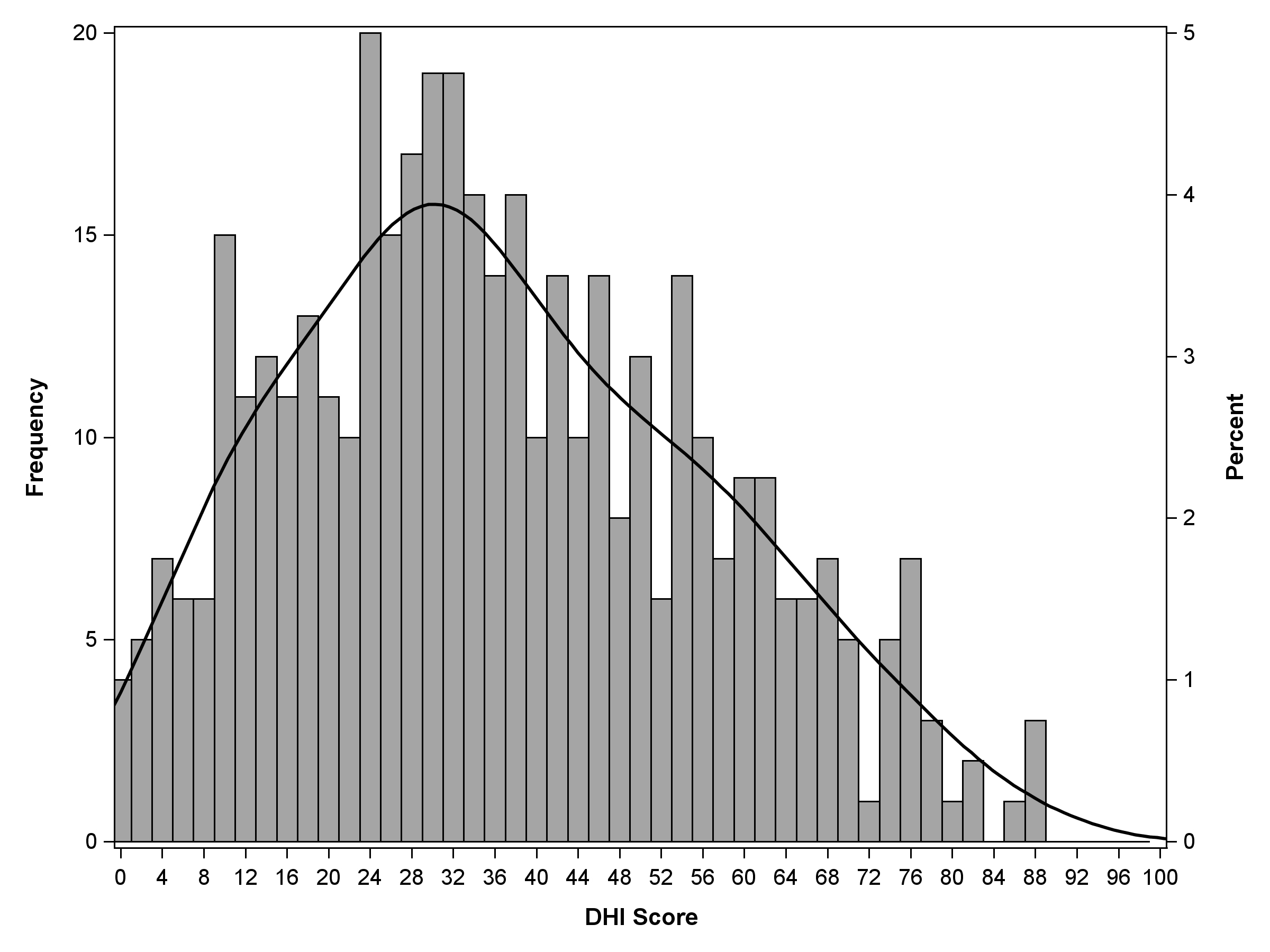

Supplement: Additional file 3 — DHI-scores and estimated kernel density curve. [file 1477-7525-9-44-S3.JPEG]
